# Galfenol Thin Films and Nanowires

**DOI:** 10.3390/s18082643

**Published:** 2018-08-12

**Authors:** Bethanie J. H. Stadler, Madhukar Reddy, Rajneeta Basantkumar, Patrick McGary, Eliot Estrine, Xiaobo Huang, Sang Yeob Sung, Liwen Tan, Jia Zou, Mazin Maqableh, Daniel Shore, Thomas Gage, Joseph Um, Matthew Hein, Anirudh Sharma

**Affiliations:** 1 Electrical and Computer Engineering, University of Minnesota, Minneapolis, MN 55455, USA; Rajneeta.Basantkumar@hotmail.com (R.B.); pmcgary@bju.edu (P.M.); eliotestrine@gmail.com (E.E.); Xiaobo.Huang@wdc.com (X.H.); neoesp@gmail.com (S.Y.S.); jiazou11@gmail.com (J.Z.); mazenakos@gmail.com (M.M.); umxxx023@umn.edu (J.U.); Heinx055@umn.edu (M.H.); shar0340@umn.edu (A.S.); 2 Chemical Engineering and Materials Science, University of Minnesota, Minneapolis, MN 55455, USA; skmadhukar@gmail.com (M.R.); liwen.tan@seagate.com (L.T.); shore033@umn.edu (D.S.); tom.e.gage@gmail.com (T.G.)

**Keywords:** Galfenol, magnetic nanowires, electrochemical deposition

## Abstract

Galfenol (Fe_1−x_Ga_x_, 10 < x < 40) may be the only smart material that can be made by electrochemical deposition which enables thick film and nanowire structures. This article reviews the deposition, characterization, and applications of Galfenol thin films and nanowires. Galfenol films have been made by sputter deposition as well as by electrochemical deposition, which can be difficult due to the insolubility of gallium. However, a stable process has been developed, using citrate complexing, a rotating disk electrode, Cu seed layers, and pulsed deposition. Galfenol thin films and nanowires have been characterized for crystal structures and magnetostriction both by our group and by collaborators. Films and nanowires have been shown to be largely polycrystalline, with magnetostrictions that are on the same order of magnitude as textured bulk Galfenol. Electrodeposited Galfenol films were made with epitaxial texture on GaAs. Galfenol nanowires have been made by electrodeposition into anodic aluminum oxide templates using similar parameters defined for films. Segmented nanowires of Galfenol/Cu have been made to provide engineered magnetic properties. Applications of Galfenol and other magnetic nanowires include microfluidic sensors, magnetic separation, cellular radio-frequency identification (RFID) tags, magnetic resonance imaging (MRI) contrast, and hyperthermia.

## 1. Introduction

In this paper, we will review 15 years of magnetic nanowire work at the University of Minnesota. In the early days, these magnetic nanowires were proposed as hair-like sensors [1], similar to the cilia found in many biological species. The material of choice was Galfenol (Fe_1−x_Ga_x_, 10 < x < 40) [2] which was a relatively new magnetostrictive alloy, rivaling Terfenol-D (Tb-Fe-Dy) with magnetostriction (430 ppm) of the same order of magnitude, yet with much more useful (ductile) mechanical properties. Where Terfenol-D is difficult to machine even coarsely (e.g., for lamination to avoid eddy currents), Galfenol can be finely machined (e.g., with small threads for suspension mounting) [3]. In fact, Galfenol *can* be used in suspension (tension), especially when stress annealing is used to produce internal compressive stresses, but brittle Terfenol-D requires the application of a compressive strain for most applications, such as sonar transducers [4,5]. This simplification of the transducer design is significant, through which entirely new designs become possible. The ductility of Galfenol also played an important role in nanowire sensors. Although Terfenol in bulk or film forms could have higher saturation magnetostrictions (up to 1600 ppm, depending on the processing conditions), it is difficult to image a process by which nanowires of Terfenol could be made without breaking. Galfenol, however, can be made into wire-like unimorphs by rolling followed by cutting [6], and even into nanowires by electrodeposition into nanoporous templates. Unlike Terfenol and other rare earth-containing alloys, a fairly wide processing window has been defined to enable the electrodeposition of Galfenol. 

The first part of this review will discuss the electrochemical processing parameters and the resulting properties of Galfenol films. Next, Galfenol nanowire synthesis and properties will be discussed. Finally, several biomedical applications of nanowires will be introduced. 

## 2. Galfenol Films and Properties

**Figure 1:**
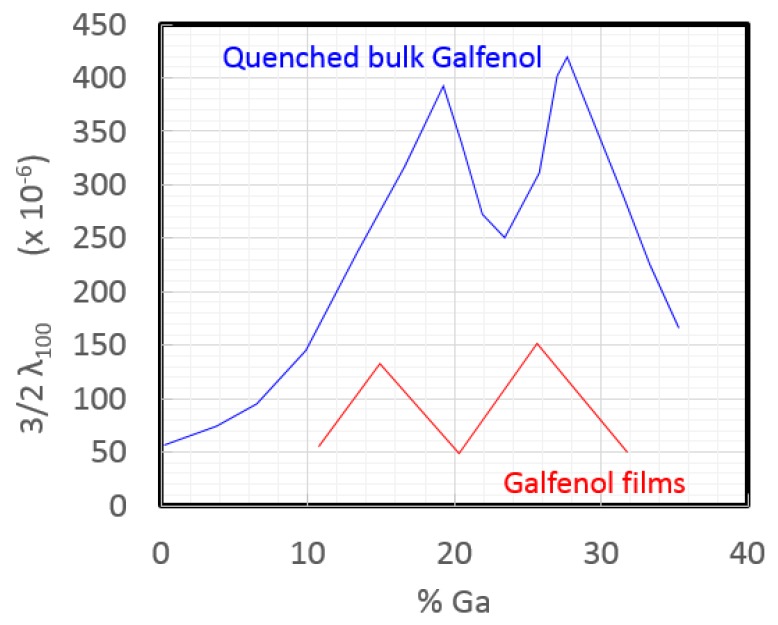
Magnetostriction vs. composition trends in bulk (blue, [7]) and sputtered films (red, [8]).

Galfenol was first studied in bulk single-crystal and polycrystalline forms at NWSC, Carderock Division in Maryland and Ames Lab in Iowa (Figure 1) [2]. The magnetostriction is largest in the (100) direction, and it has a bimodal dependence on Ga composition with 400 ppm at 19% Ga and 430 ppm at 25% Ga, where both require optimized quenching [7]. To produce thin films of Galfenol, sputtering was attempted by Basantkumar at the University of Minnesota [8] using sputtering targets provided by Etrema, Inc. in Iowa. The magnetostriction of these films, measured using a capacitance bridge technique, also appeared to have a bimodal dependence on Ga composition, although shifted from bulk values due to the state of strain in the films with a maximum value nearing 150 ppm, Figure 1 [7,8]. This means that the ideal composition shifted from 19% to 15% Ga for the lower peak, but 25% Ga also remains a good composition even with strain. Although not as large as bulk magnetostriction values, 150 ppm is sufficient for new microelectromechanical system (MEMS) cantilever sensors made possible by the formation of the film. 

Thicker films (~microns) are preferred for many applications but are difficult to achieve with vacuum fabrication, so electrodeposition was explored by our group and others [9,10,11]. The processing window for Galfenol spanned a wide range of current densities and aqueous electrolyte compositions (Table 1). In our group, this was accomplished using a combinatorial technique in a Hull cell, which is a trapezoidal electrochemical cell where the current density (*j*) during deposition varies as a function of total current (*I*) and distance along the working electrode (*x*), according to Reference [12]:

*j*(*x*) = *I* (51.04 − 52.42 × log *x*) for 0.635 < *x* < 8.255 cm. (1)

**Figure 2:**
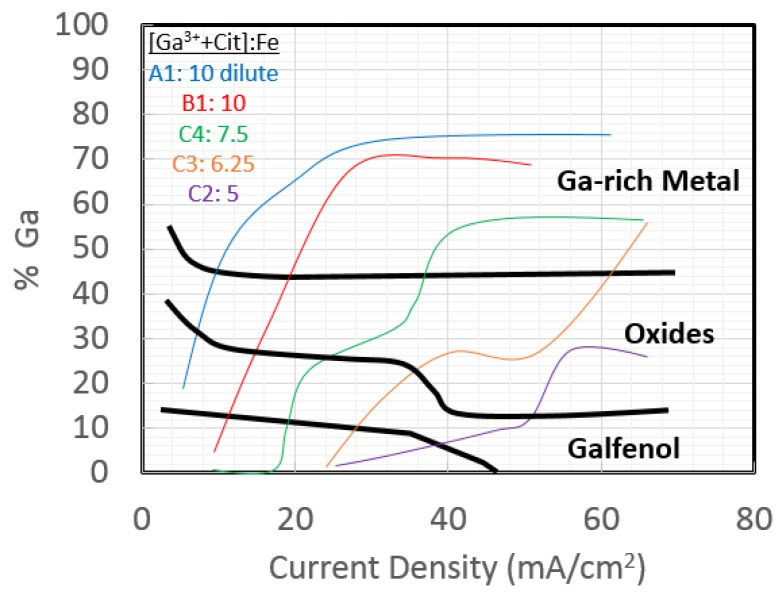
Electrochemical phase diagram showing the regions of ion ratios and current densities that produce Galfenol or other phases. Electrolyte ion concentrations are shown in the legend [11].

**Figure 3:**
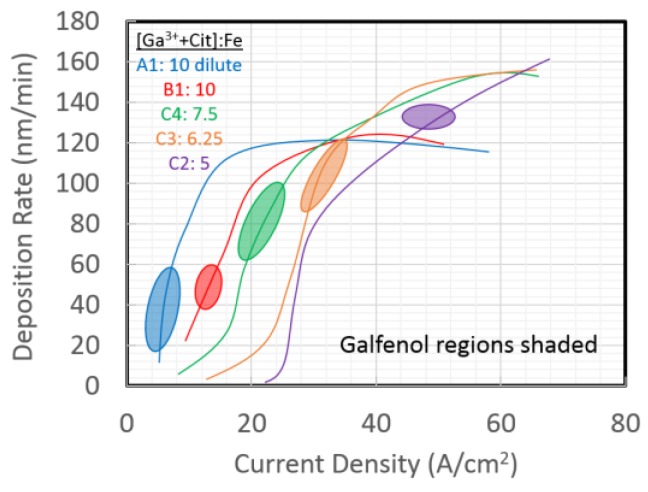
Deposition rates for films from electrolytes that produced Galfenol (shaded regions). Electrolyte ion concentrations are shown in the legend [11].

It was difficult to establish a direct trend between Ga^3+^ content in the electrolyte and %Ga in the resulting films. However, through the addition of sodium citrate to the electrolyte, Ga^3+^ formed a complex with citrate (Cit) anions, and a trend was established for the dependence of %Ga in the films. The important relationship is the ratio of the Fe^2+^ concentration [Fe^2+^] vs. the sum of [Ga^3+^] *and* [Cit] in an electrolyte, as shown in the “phase diagram” of Figure 2. The deposited films were segregated into regions defined as Fe metal, Galfenol, oxides, and Ga-rich metal. Results are shown only for electrolytes that produced a Galfenol region. As expected, the deposition rates increased as a function of current density, Figure 3, until they reached a saturation value. A novel energy dispersive spectroscopy technique was used to quantify the composition and thickness of films, and these results were verified by Rutherford backscattering (RBS). 

**Figure 4:**
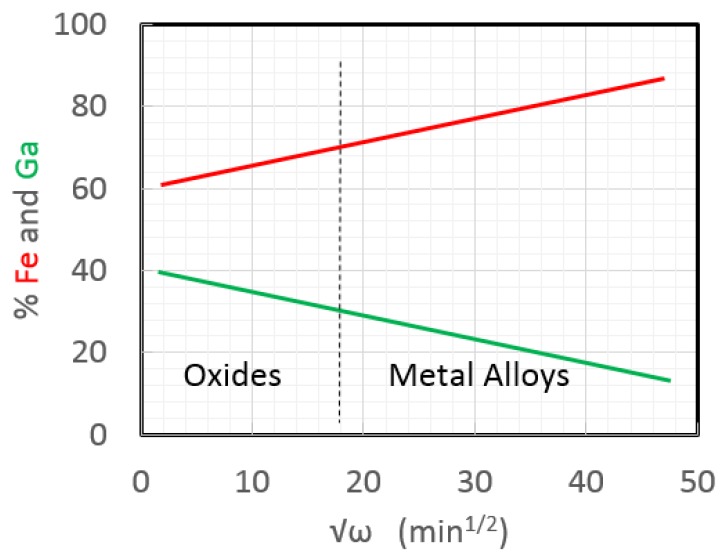
Due to differences in mass transport rates, the composition of Galfenol films can be tailored simply using the rotation rate (*ω*) of a rotation disk electrode [14].

Non-combinatorial studies further increased the understanding of Gafenol electrodeposition. First, Reddy [13] found that it was best to make electrolytes by adding sodium citrate, sodium sulfate, iron sulfate, and gallium sulfate, in this order. With similar concentrations to prior studies, Reddy then used a rotating disk electrode to control the diffusion boundary layer in the electrolyte to achieve steady-state electrochemistry due to parallel processes of mass transfer and kinetics. The mass transfer-limited current densities increased with the square root of the electrode rotation (*ω*), owing to a thinning boundary layer. The kinetic proportionality constant was dependent on the number of electrons taking part in the overall reaction, the Faraday constant, the bulk concentration of the cations, the ion diffusivity in the electrolyte, and the kinematic viscosity of the electrolyte. This balance of processes is sometimes called the Koutecky-Levich relationship. Reddy used fixed potentials to obtain similar current densities as prior combinatorial studies, thereby determining the kinetic-limited current density at each voltage using a Tafel plot [14]. A mechanism was proposed based on Reference [15] in which an absorbed monovalent [*Fe(I)*]*_ads_* intermediate is the rate-determining first step, Equation (2). This intermediate then either reduces to a Fe (solid) deposit, or catalyzes Ga (solid) via an adsorbed [*Ga(III)* − *Fe(II)*]_ads_ intermediate (Equations (3)–(5)). Since Fe^2+^ and Ga^3+^ have different mass-transport rates, the composition can be controlled simply by varying the rotation rate (*ω*), as shown in Figure 4.

(2) $$Fe\left(II\right)+e^{-}\to {\left[Fe\left(I\right)\right]}_{ads}$$

(3) $${\left[Fe\left(I\right)\right]}_{ads}+e^{-}\to Fe\left(s\right)$$

(4) $${\left[Fe\left(I\right)\right]}_{ads}+Ga\left(III\right)\to {\left[Ga\left(III\right)-Fe\left(I\right)\right]}_{ads}$$

(5) $${\left[Ga\left(III\right)-Fe\left(I\right)\right]}_{ads}+3e^{-}\to Ga\left(s\right)+{\left[Fe\left(I\right)\right]}_{ads}$$

**Figure 5:**
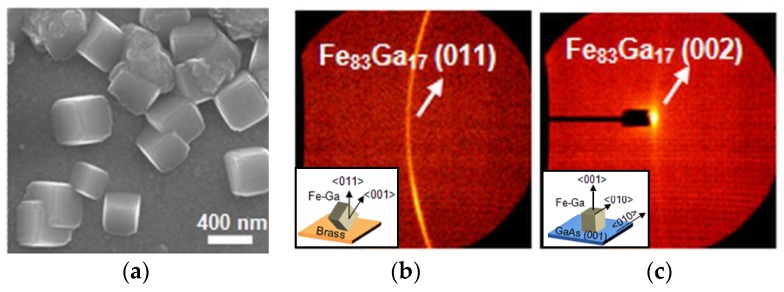
Galfenol grown with slow deposition rates onto (**a**) brass substrates, resulting in (**b**) primarily cube on edge texturing. (**c**) Although some grains were observed with low-energy (110) orientations, a majority of the grains were epitaxially oriented on GaAs. Reproduced from [13], with the permission of AIP Publishing.

Our next goal was to orient the Galfenol films to have the maximum magnetostriction, namely (100) texture, using n-GaAs as a substrate for epitaxial growth [13]. Reddy used both brass substrates (similar to Hull cell and rotating disk studies) and epi-ready n-GaAs substrates (2° miscut, 2 × 10^17^ cm^−3^ Si-doping). A slower growth rate was used than in the previous study to enable better epitaxy; as a result, some nuclei had time to grow with low-energy (110) surfaces on both substrates. Therefore, on the brass substrate, the texture parallel to the surface was (110) and (211) as before (Figure 5a,b). On GaAs, however, epitaxy in the (100) planes was also achieved, as seen in the areal X-ray diffraction pattern in Figure 5c. Rocking curves verified these results [13].

**Figure 6:**
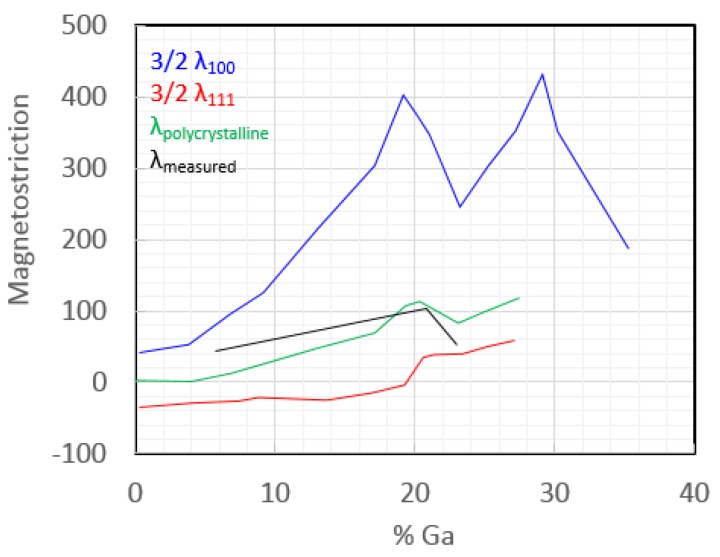
Measured values for the magnetostriction of Galfenol crystallographic orientations and the calculated value of polycrystalline Galfenol, which compares well to measured values [17].

The capacitance bridge measurements of sputtered Galfenol magnetostriction required thin, well-characterized, insulating substrates, such as glass coverslips, but electrodeposited Galfenol films require conductive substrates, mostly metals. Estrine showed that sputter-deposited Cr/Cu adhesion layers were effective contacts for the deposition of Galfenol films (170 nm) onto glass coverslips [16]. Magnetostriction was measured as high as 140 ppm for these 17–21% Ga films. This was the first time that electrodeposited Galfenol was confirmed to be magnetostrictive. The next year, Estrine measured the values at a variety of compositions [17] and found that the values agreed with what would be expected for polycrystalline Galfenol (Figure 6). 

A summary for the study of electrodeposition for Galfenol is that films can be engineered to have composition, crystal structure, and magnetostriction sufficient for microelectromechanical system (MEMS) devices. In fact, a recent visitor to our lab, Vargas, demonstrated that Galfenol films are also biocompatible by internalizing Galfenol-coated discs into cells [18]. Additionally, increasing interest in bio applications, such as the initially proposed cilia sensors, led this work toward the electrochemical synthesis of nanowires, which is presented next.

## 3. Galfenol Nanowires and Properties

**Figure 7:**
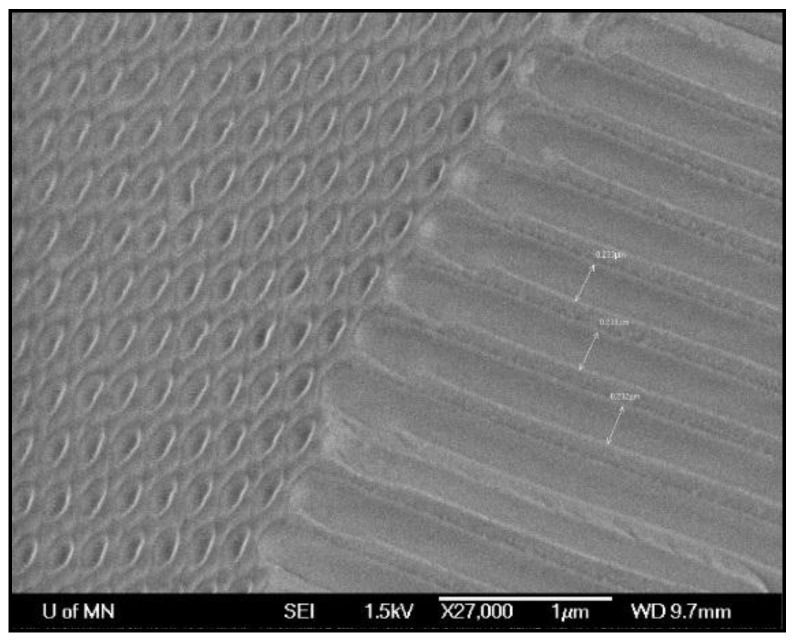
Anodic aluminum oxide template [19].

To make nanowires, an electrically conductive film (e.g., Ti/Cu or W/Cu) is sputter-deposited onto one side of a nanoporous anodic aluminum oxide (AAO) template (Figure 7) [19]. The coated side is insulated with a polymer coating so that when the AAO is placed inside an electrolyte such as those discussed above, the cations are reduced to metal only at the bottom of the columnar nanopores. This reduced metal deposit grows inside the template, conforming to the shape of the nanopores and resulting in nanowires. 

**Figure 8:**
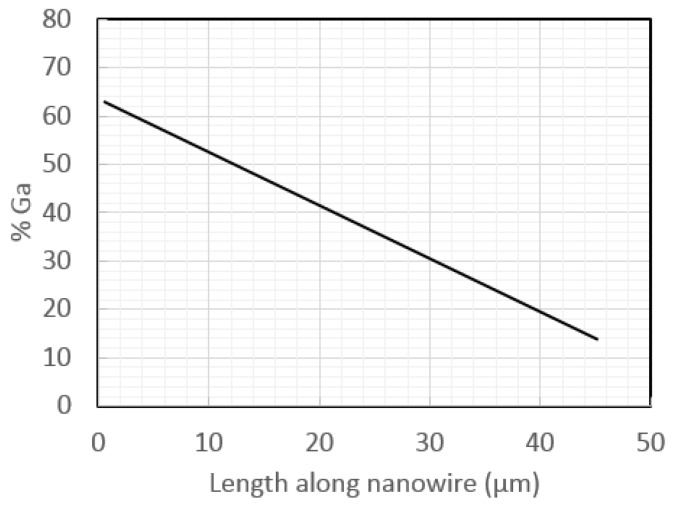
Initial trend of compostion change along nanowire lengths, later mitigated using [Cit] complexing [9].

**Figure 9:**
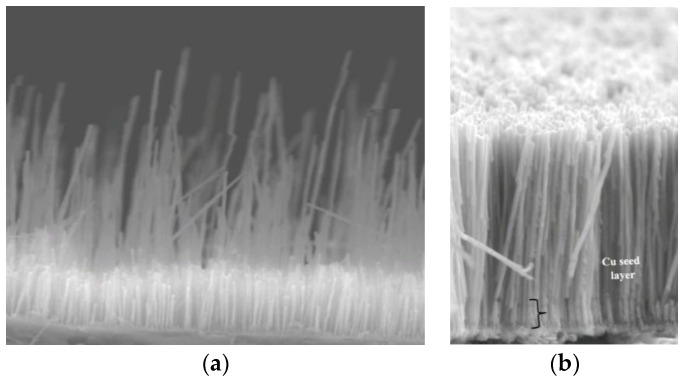
(**a**) A second problem unique to Galfenol was extremely inhomogeneous lengths, where many short nanowires (~4 μm) were seen at the growth electrode while others grew extremely fast, even to the top of the template. (**b**) Uniform lengths (8 μm ± 3%) were achieved using a rotating disk electrode, a Cu seed layer, and pulsed deposition [20].

In initial nanowire studies, the Fe:Ga composition varied significantly along the length of the nanowires (Figure 8) [9]. This was believed to be due to a decreasing distance between the counter electrode and the working electrode, which was the growing ends of the nanowires. A [Cit] complexing agent was discovered that helped stabilize the Ga in solution, and therefore mitigated the composition variations [11]. However, highly variable lengths were often seen in Galfenol nanowire arrays to a degree that was orders of magnitude more severe than that of other metal nanowires. Modeling showed that hemi-spherical diffusion boundary layers caused a few of the nanopores to ‘steal’ cations from the nanopores around them [20], resulting in the strange bi-modal lengths seen by SEM in Figure 9a. Reddy applied the rotating disk electrode that was used in his steady-state film electrochemistry to keep the diffusion boundary layer planar (not hemi-spherical). This technique, together with Cu seed layer growth prior to Galfenol growth and pulsed electrodeposition, led to nanowire lengths with only 3% standard deviation, as shown in Figure 9b. It is important to mention that this severe inhomogeneity in nanowire length was unique to Galfenol and did not occur in any other metal alloys deposited in our group to date.

Once reproducible Galfenol nanowires were available, three new application-related questions revealed themselves. Can nanowires be ordered with controllable spacings? Can nanowires be integrated with useful substrates, such as Si? What can be done about shape anisotropy so that magnetostriction can be utilized?

**Figure 10:**
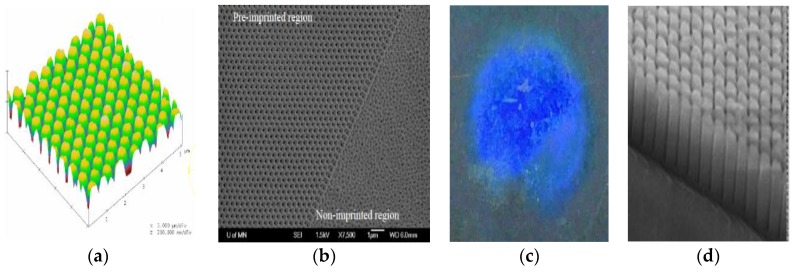
(**a**) Si_3_N_4_ nanostamp to imprint Al films and foils so that (**b**) nanopores aligned in the subsequent oxide formed by anodization of the Al. (**c**) Line stamps of Ni could be made large enough to use for large area (1 cm in diameter) alignment, shown diffracting blue light. (**d**) Magnification of nanopores (1 μm long) produced by line stamps [23,26].

First, controllable nanowire size/spacing can be made by imprinting precursor Al before it is anodized to produce the nanoporous oxide template, Figure 10. Al is a relatively soft material, so nanostamps can be made of many other materials [21]. Tan [22] used e-beam defined Si_3_N_4_ on Si, Figure 10a, and found that rounded pillars can imprint Al well enough (20 nm dimples) to enable nanopores to align with the imprint upon anodization, as shown in Figure 10b [21,23]. These pillars were more manufacturable than the pointed stamps that were assumed to be required by the literature at the time [24,25]. Figure 10b shows nanopores that formed at a boundary of stamped and unstamped Al after subsequent oxidization via anodization. Large areas can be difficult to obtain if a stamp is made by e-beam lithography, especially if very small pillars/spacings are desired. For example, in 2009, a 4 square inch nanostamp of hexagonally spaced (30 nm) pillars would take 23 years of ebeam writing! Today, e-beams can translate between written areas faster, but our current design is more novel and faster than pillars. Specifically, line stamps are imprinted into the Al precursor twice with 60 degrees between imprints to produce directed self-assembly of hexagonally close-packed nanopores when the Al is oxidized [26]. This technique can be used for areas on the order of square inches with little time or cost, as depicted in Figure 10c,d [26].

The second applications-related question was whether electroplated nanowires could be integrated with convenient platforms, such as Si, for use in electronic, spintronic, photonic, MEMS/NEMS (micro/nano electromechanical systems), microfluidics, or other systems. Most early nanowire AAO papers involved four steps: anodizing Al foils, removing remnant Al, etching the oxide off of the bottom of pores, and finally growing nanowires. Zhou [23] found that Al films could be evaporated onto Ti/Cu-coated Si for subsequent anodization, and that these films could be nanoimprinted to enable ordered nanowire arrays to be integrated with Si. If the anodization is carried out until the Al is fully consumed, the barrier can be removed electrochemically to enable nanowire growth into the pores using the original Ti-Cu undercoating. Zhou also etched into the Si to form divots for subsequent ordered nanoporous Si. Huang [27] and then Maqableh [28] used this anodization to integrate 10 nm diameter spintronic nanowires inside AAO on Si. Interestingly, the sidewalls of the AAO nanopores were so smooth that the Cu nanowires grown inside of them had almost bulk resistivity, despite having diameters 4× smaller than the mean free path of the electrons [28].

**Figure 11:**
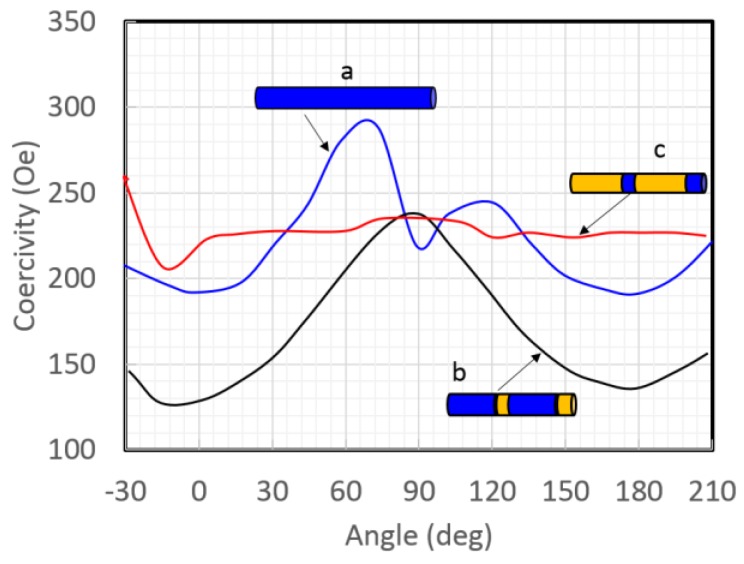
The coercivity from magnetic hysteresis loops for (**a**) pure Galfenol nanowires and segmented Galfenol/Cu nanowires with (**b**) 50 nm of Cu in Galfenol nanowires or (**c**) 50 nm Galfenol in Cu nanowires (diameter = 100 nm; long segments 400 and 500 nm, respectively).

Third, to actively use magnetostriction, the effect must dominate over shape anisotropy, which is strongly oriented along the nanowire axes due to the very high aspect ratios (length/width = 10–200 nm/1–10 μm). By definition, magnetostriction is a change in dimension due to an applied magnetic field. The reverse phenomenon can be useful as well; namely, a magnetization change can be measured when the material is strained. In single-component magnetic nanowires, the magnetization cannot rotate in response to an applied force or vibration due to the shape anisotropy (2πM_s_, where M_s_ is the saturation magnetization). To get around this constraint, segmented nanowires were made, typically FeGa/Cu [20]. Hysteresis loops taken at varied angles reveal the reversal mechanism of the magnetization in these nanowires. For example, the 100 nm diameter samples depicted in Figure 11a [20] mostly reverse by vortices, characterized by a minimum coercivity (H_c_) when the applied field is parallel to the nanowire axes and a maximum coercivity when the applied field is perpendicular. The severe shape anisotropy of the single component nanowires (curve “a”) caused a deviation from this behavior, such that the magnetization coherently rotated after perpendicular saturation, leading to a lower H_c_ at that angle. Sample “b” exhibited classic behavior. Importantly, sample “c” had an isotropic magnetic signature since the Galfenol segments were isotropic in shape, and they were separated from each other by long Cu segments which minimized dipolar interactions. This isotropic sample is most likely to exhibit a change in magnetization if strained or a change in dimension if magnetized.

During most of our Galfenol nanowire studies, we collaborated with Prof. Alison Flatau’s group at the University of Maryland. Downey used a nanomanipulator to measure the Young’s modulus of Galfenol nanowires to be about 58 GPa by tensile testing [29]. These values are promising in that ductile behavior is needed for many of the bio and sensing applications where these nanowires will essentially be used as nanoelectromechanical systems (NEMS). Park used magnetic force microscopy (MFM) [30] to show that the GaFe segments in GaFe/Cu nanowires exhibited vortices at remanence, but the moment was fully parallel to the nanowire when fields larger than 300 Oe were applied in this direction. Park was also able to measure the hysteresis loops [31] and magnetostriction [32] of single nanowires using atomic force microscopy (AFM) techniques. Finally, Park used in Galfenol/Cu segmented nanowires to demonstrate that they could be used as pressure sensors, where pressure was applied to strain the nanowires. This induced a magnetization change in the nanowires that was detected with giant magnetoresistive (GMR) film sensors below the nanowire array [33].

Newer collaborations include those at National Institute of Standards and Technology (NIST), Gaithersburg, where Grutter et al. used polarization-analyzed small angle neutron scattering (PASANS) to observe complex three-dimensional ordering between Galfenol segments in FeGa/Cu nanowires [34]. Combined dipolar interwire and intersegment interactions led to ordering that was ferromagnetic between nanowires and antiferromagnetic between segments parallel to the nanowires. Ponce et al. from the University of Texas, San Antonio used holographic TEM to also observe vortex and antiferromagnetic ordering in thin layers (1–2 nanowires thick) [35].

## 4. Bio Applications: Flow Sensors, Purification, Barcodes, RFID Nanotags, and MRI Contrast

**Figure 12:**
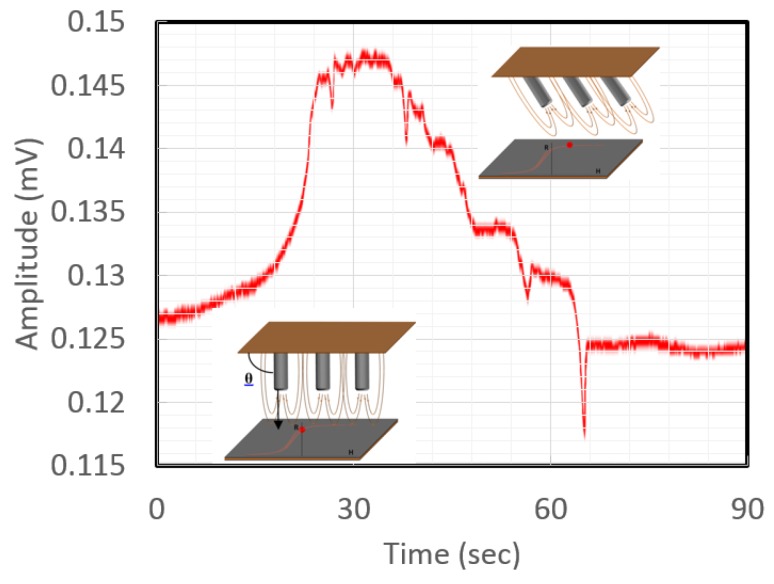
Signal from magnetoresistive sensors at the bottom of a microfluidic channel showing a direct proportionality to flow due to a rapid increase in pressure then a slow release with three sudden drops in pressure and a final backwash from complete pressure release [36].

Our first biosensor used cobalt (Co). nanowires and a magnetoresistive sensor at the bottom of a microfluidic channel. Hein et al. detected flows in microfluidic channels from Diagnostic Biosensors that varied from 0.5 mL/min to 6 mL/min with a signal-to-noise ratio (SNR) of 44 using only 140 μW of power and no amplification, Figure 12 [36]. The magnetoresistive sensors were sensitive to “in-plane” moments that were provided by the nanowires as they bent in the flow, as shown. Vibration sensors were also tested using these nanowires. 

Sharma et al. incubated Au-tipped Ni nanowires with osteosarcoma for our first look at internalization via endosomes [37], which was later confirmed with a more rigorous study that involved PEG and peptide (RGD) coatings [38]. These nanowires were found to have low cytotoxicity, first by acridine orange and propidium iodide dual staining [37] and later by IL-1*β*, TNF-*α*, and MTS assays. It is also worth mentioning that although these Au-tipped nanowires showed very low cytotoxicity, the Au was deposited from a cyanide-based electrolyte. A thiosulfate-sulfite electrolyte was developed for Au deposition by Estrine and Tabokovic et al. [39] as an alternative to cyanide. Interestingly, the integrin overexpression in osteosarcoma led these cells to self-dispersal of RGD-coated nanowires by attachment and motility, followed by internalization and propagation via cell splitting and proliferation. Cells with internalized nanowires could be purified from their assays via magnetic separation [40]. 

Results such as the ones shown in Figure 11 indicated that it could be possible to barcode cells using nanowires with distinct magnetic signatures. For example, samples a, b, and c in Figure 11 have fairly distinct signatures. By varying diameter as well as segmentation, even more barcodes are possible since coercivity vs. angle curves are highly dependent on magnetization reversal mechanisms. Reddy found that 35 nm FeGa nanowires appear to have diameters that are too small to support vortices, leading to new magnetic signatures [40]. 

Although coercivity vs. angle appears to contain distinguishing characteristics for different nanowires, normal means of measuring the hysteresis loops, e.g., vibrating sample magnetometry, cannot easily distinguish mixtures of barcode nanowires. Sharma demonstrated the use of first-order reversal curves to demultiplex mixtures [41]. In recent collaborations with Prof. Rhonda Franklin, we are exploring options to use high frequency (e.g., radio frequency) identification (RFID) methods to distinguish mixtures, an area in which Zhou and Um have recently presented promising results [42,43]. Conveniently, this research has cross-fertilizing potential, and Zhou designed the first coplanar waveguide circulator [44].

Many other bio applications are under study by our group and others because magnetic nanowires have potential in magnetic resonance imaging (MRI) contrast [45], targeted cell separation, and hyperthermia. Magnetic nanowires have high saturation magnetizations and high shape anisotropy compared to superparamagnetic iron oxide nanoparticles (SPIONs), which have become ubiquitous in bio-magnetic studies. Besides their magnetic advantages, these nanowires have larger surface areas for attaching biomolecules. New literature is sure to make this review article dated upon publication, thanks to an exciting frontier ahead.

## 5. Conclusions

Thin films and nanowires of Galfenol have been made and studied in Minnesota for several years. Beginning with sputter deposition to verify thin film properties, our group moved on to define a phase diagram for the electrochemical deposition of this important magnetostrictive alloy. To keep Gallium available to deposit, a citrate complexing agent was identified. To control the composition, a rotating disk electrode (RDE) was used to engineer the diffusion boundary layer. This same RDE was instrumental in mitigating a problem of severely inhomogeneous lengths that was unique to Galfenol nanowire growth. Cu seed layers and pulsing can be added to RDE if absolute length uniformity is required for a given application. The properties of Galfenol films have been extensively studied, showing that they are polycrystalline with the expected magnetostriction for this cubic alloy and that they can be epitaxially deposited on GaAs. Galfenol nanowires were made by electrodeposition into anodic aluminum oxide (AAO) templates. These templates were made with long range order for the careful placement of nanowires in arrays, both as free-standing samples and as integrated components on Si. Segmentation of Galfenol nanowires with Cu enabled controlled magnetic properties, including isotropy magnetic shapes. Several bio applications of magnetic nanowires have been published, including microfluidic sensors and MRI contrast agents, and others are on the immediate horizon, including magnetic separation, cellular RFID tags, and hyperthermia.

## Figures and Tables

**Table 1:** Electrolytes used in combinatorial study.

ID	Fe^2+^:Ga^3+^ Ion Ratio	[Fe_2_SO_4_] (M)	[Ga_2_(SO_4_)_3_] (M)	Na-Cit (M)
A1	1:5	0.01	0.025	0.05
B1	1:5	0.02	0.05	0.1
B2	1:2.5	0.04	0.05	0.1
C2	1:2.5	0.04	0.05	0.1
C3	1:2.5	0.04	0.05	0.15
C4	1:2.5	0.04	0.05	0.2
